# Supplementary material for: Radioactive contamination in feral dogs in the Chernobyl exclusion zone: Population body-burden survey and implications for human radiation exposure
Source: PLoS One. 2023 Jul 20;18(7):e0283206. doi: 10.1371/journal.pone.0283206 (PMC10358910; doi:10.1371/journal.pone.0283206)
Supplement: Attachment — Submitted filename: Response Letter.pdf [file pone.0283206.s002.pdf]

Editor Responses

We thank the editors for their patience and support with our submission. Please find our responses below in red.

1. Please ensure that your manuscript meets PLOS ONE's style requirements, including those for file naming. The PLOS ONE style templates can be found at

https://journals.plos.org/plosone/s/file?id=wjVg/PLOSOne_formatting_sample_main_body.pdf and

https://journals.plos.org/plosone/s/file?id=ba62/PLOSOne_formatting_sample_title_authors_affiliations.pdf

We have updated our manuscript to reflect the style guidelines outlined. Our manuscript was written in Latex, and we have also included the Latex source files in our newest resubmission. Please find that we have separated the abstract and author summary from the title page. We have also amended our title to fit with the sentence case structure.

2. In your Methods section, please provide additional information regarding the permits you obtained for the work. Please ensure you have included the full name of the authority that approved the field site access and, if no permits were required, a brief statement explaining why.

We thank the editor for pointing this out. We have updated our manuscript and associated github with the requested information and materials.

3. Please update your submission to use the PLOS LaTeX template. The template and more information on our requirements for LaTeX submissions can be found at http://journals.plos.org/plosone/s/latex.

Both our original and most recent submission have leveraged the provided Latex template. We downloaded the template and used it to write our manuscript in Overleaf. If we are using something in error, please let us know.

4. We note that Figure 1 in your submission contain [map/satellite] images which may be copyrighted. All PLOS content is published under the Creative Commons Attribution License (CC BY 4.0), which means that the manuscript, images, and Supporting Information files will be freely available online, and any third party is permitted to access, download, copy, distribute, and use these materials in any way, even commercially, with proper attribution. For these

reasons, we cannot publish previously copyrighted maps or satellite images created using proprietary data, such as Google software (Google Maps, Street View, and Earth). For more information, see our copyright guidelines: http://journals.plos.org/plosone/s/licenses-and-copyright.

We require you to either (1) present written permission from the copyright holder to publish these figures specifically under the CC BY 4.0 license, or (2) remove the figures from your submission:

a. You may seek permission from the original copyright holder of Figure 1 to publish the content specifically under the CC BY 4.0 license.

We recommend that you contact the original copyright holder with the Content Permission Form (http://journals.plos.org/plosone/s/file?id=7c09/content-permission-form.pdf) and the following text:

“I request permission for the open-access journal PLOS ONE to publish XXX under the Creative Commons Attribution License (CCAL) CC BY 4.0 (http://creativecommons.org/licenses/by/4.0/). Please be aware that this license allows unrestricted use and distribution, even commercially, by third parties. Please reply and provide explicit written permission to publish XXX under a CC BY license and complete the attached form.”

Please upload the completed Content Permission Form or other proof of granted permissions as an "Other" file with your submission.

In the figure caption of the copyrighted figure, please include the following text: “Reprinted from [ref] under a CC BY license, with permission from [name of publisher], original copyright [original copyright year].”

b. If you are unable to obtain permission from the original copyright holder to publish these figures under the CC BY 4.0 license or if the copyright holder’s requirements are incompatible with the CC BY 4.0 license, please either i) remove the figure or ii) supply a replacement figure that complies with the CC BY 4.0 license. Please check copyright information on all replacement figures and update the figure caption with source information. If applicable, please specify in the figure caption text when a figure is similar but not identical to the original image and is therefore for illustrative purposes only.

The following resources for replacing copyrighted map figures may be helpful:

USGS National Map Viewer (public domain): http://viewer.nationalmap.gov/viewer/

The Gateway to Astronaut Photography of Earth (public domain): http://eol.jsc.nasa.gov/sseop/clickmap/

Maps at the CIA (public domain):
https://www.cia.gov/library/publications/the-world-factbook/index.html and
https://www.cia.gov/library/publications/cia-maps-publications/index.html

NASA Earth Observatory (public domain): http://earthobservatory.nasa.gov/

Landsat: http://landsat.visibleearth.nasa.gov/

USGS EROS (Earth Resources Observatory and Science (EROS) Center) (public domain):
http://eros.usgs.gov/#

The map visualizations shown in Figure 1 our of manuscript were created using the Harvard Map Collection and The MAPA: Digital Atlas of Ukraine program. The basis for this program is NASA. To our knowledge, the images in Figure 1 are under creative commons license (CC BY 4.0) and should be usable.

5. We note that Figure 3a in your submission contain copyrighted images. All PLOS content is published under the Creative Commons Attribution License (CC BY 4.0), which means that the manuscript, images, and Supporting Information files will be freely available online, and any third party is permitted to access, download, copy, distribute, and use these materials in any way, even commercially, with proper attribution. For more information, see our copyright guidelines: http://journals.plos.org/plosone/s/licenses-and-copyright.

We require you to either (1) present written permission from the copyright holder to publish these figures specifically under the CC BY 4.0 license, or (2) remove the figures from your submission:

a. You may seek permission from the original copyright holder of Figure 3a to publish the content specifically under the CC BY 4.0 license.

We recommend that you contact the original copyright holder with the Content Permission Form (http://journals.plos.org/plosone/s/file?id=7c09/content-permission-form.pdf) and the following text:

"I request permission for the open-access journal PLOS ONE to publish XXX under the Creative Commons Attribution License (CCAL) CC BY 4.0 (http://creativecommons.org/licenses/by/4.0/). Please be aware that this license allows unrestricted use and distribution, even commercially, by

third parties. Please reply and provide explicit written permission to publish XXX under a CC BY license and complete the attached form."

Please upload the completed Content Permission Form or other proof of granted permissions as an "Other" file with your submission.

In the figure caption of the copyrighted figure, please include the following text: "Reprinted from [ref] under a CC BY license, with permission from [name of publisher], original copyright [original copyright year]."

b. If you are unable to obtain permission from the original copyright holder to publish these figures under the CC BY 4.0 license or if the copyright holder's requirements are incompatible with the CC BY 4.0 license, please either i) remove the figure or ii) supply a replacement figure that complies with the CC BY 4.0 license. Please check copyright information on all replacement figures and update the figure caption with source information. If applicable, please specify in the figure caption text when a figure is similar but not identical to the original image and is therefore for illustrative purposes only.

We thank the editor for their note and we take great pride in the quality of the graphic featured in Figure 3a. The graphic used in Figure 3a was created by a member of one of the co-authors laboratory – specifically for use in the paper. This graphic has no current copyright. The graphic artist who provided the graphic is acknowledged in the manuscript.

6. Please review your reference list to ensure that it is complete and correct. If you have cited papers that have been retracted, please include the rationale for doing so in the manuscript text, or remove these references and replace them with relevant current references. Any changes to the reference list should be mentioned in the rebuttal letter that accompanies your revised manuscript. If you need to cite a retracted article, indicate the article's retracted status in the References list and also include a citation and full reference for the retraction notice.

We thank the editor for their note for this note. We have checked our references and we do not believe we have cited any retracted papers. However, we have altered the manuscript references and removed a number of problematic citations. This does not change the correctness of our current citation usage. We have updated our references in accordance with feedback from the reviewers.

Review of Hecla et al. (Radioactive Contamination in Feral Dogs in the Chernobyl Exclusion Zone: Population Body-Burden Survey and Implications for Human Radiation Exposure)

Page	line	Comment
	Abstract	Even high levels of contamination rarely pose a health risk, even to those who are contaminated. Did the authors quantify the amount of contamination present on any of the dogs, calculate the amount that could reasonably transfer to a person, and determine the potential health effects of this transfer? Barring some sort of calculation to this effect, I have to confess to being somewhat dubious about the potential for external contamination on a dog to transfer to a person in sufficient quantity to pose a health risk.<br>- The quantification of the total surface activity is in the results section, and has been amended to reflect uncertainty in transfer coefficients . While the dose caused by ingesting or inhaling this quantity of material is not large in absolute terms, it is significant above exposure thresholds in place for members of the public. We have edited the paper to reflect this
3	30	Again, I am somewhat dubious about contamination posing a health risk, even if transferred to skin. Can the authors provide some calculations based on average and maximum removeable contamination levels they found, indicating a plausible dose to the skin from incidental contact with contaminated animals, and to the whole body in the event that some of this contamination were to transfer to food and be ingested? I understand that, according to ALARA, any exposure is to be avoided if possible – but I'm also guessing that tourists and animals are likely to be unfamiliar with the ALARA principle , so such an assessment seems reasonable.<br><br>-We have added this analysis to the results section. We appreciate these edits, as they significantly improve the paper.

4	34	Similarly, can the authors calculate exposure to people at a distance of, say, 30 cm and 1 m from the contaminated animals they measured? How does the calculated dose rate compare to the actual dose rates measured?<br>-Due to the fact the emitters (the dogs) have a spatial extent comparable to the standoff distance, simple inverse square scaling does not work. As a result, we would have to run detailed particle transport calculations to answer this question properly. As the doserates are negligible for external exposure, we have modified the language to reflect the minimal nature of the hazard.
4	75	How does the background noted here compare to unshielded background count rate outside at this location?<br>-This has been added in. It's about a factor of ten reduction.
5	96	Do the authors have a citation for the high counting efficiency noted here? It's not that I doubt them – just that Knoll notes an intrinsic counting efficiency of a little less than 90% for a 4" NaI detector and, when we add to that a geometric counting efficiency of 50% or less, the absolute counting efficiency is more likely around 40-45% for Cs-137.<br>-This was phrased poorly. We were referring to the intrinsic efficiency, not the overall counting efficiency. This has been revised to clarify.
5	100	Good call on not using K-40! The amount in concrete (and brick, for that matter) depends strongly on the clay mineralogy of these materials<br>-Thank you. We made this call after attempting this in the lab– the corrections just didn't work well.
6	131	Can the authors provide a reference (or calculation) for 22% counting efficiency for Sr-90? Also, is this for Sr-90 alone, or are you assuming that Y-90 is in equilibrium? If Y-90 is present, the actual counting efficiency might be much higher due to the presence of the second beta and its much higher energy.<br><br>-This is from the user's manual for the Ludlum 26-2, and assumes the Y90 is in equilibrium. You are correct to point out this, and we have corrected our values to reflect an equilibrium Sr90/Y90 concentration (https://ludlums.com/images/data_sheets/M26-2.pdf) We have also included this reference in the manuscript.

7	160	How did the authors differentiate between the beta and gamma radiation when determining the counting efficiency? Or is the gamma counting efficiency simply too low to be a factor (in which case, perhaps this can be noted)?<br>-Gamma efficiency is extremely low (<1%) for the sensor used. Further, a crude gamma/beta discrimination test (flipping the pancake to irradiate the tube from the back, which is shielded by 5mm Fe) was used on all contaminated animals to determine if significant gamma emitter contamination was present.
7	168	Here, too, calculated exposure rates would be helpful. We have addressed this in the text. Thank you for pointing this out.
8	190	Do the authors recommend that anyone coming into physical contact with animals wear gloves, wash their hands, shower, change clothing, etc afterwards to reduce their exposure?<br>We have added this recommendation.
		Can the authors suggest mechanisms by which touching a contaminated animal can result in significant internal contamination and likely exposures? I can think of a few (e.g. eating a sandwich before washing one's hands) – but this seems unlikely to transfer sufficient contamination to produce a high internal exposure.<br>We noted multiple incidents of transfer to the hands and clothes of workers in the clinic requiring decontamination. Notes from the 2017 campaign indicate at least a half-dozen incidents of volunteers requiring clothes be changed or otherwise decontaminated due to contact with the dogs.
8	200	The paper mentions using an "energy-compensated NaI(Tl) detector. I am familiar with using energy-compensated GM detectors and have used many of them over the years. But I have never heard the term applied to sodium iodide. Can the authors indicate which of the NaI(Tl) detectors mentioned was energy-compensated?<br><br>-This was an error, thank you for pointing this out. The doserate function in the SAM940 uses the gamma ray energy to determine doserate. However, this is not true "energy compensation," and this has been removed.

8	201	Thanks for noting that 1 μSv/hr is not a very high dose rate – this might be worth mentioning in the abstract as well, even if only qualitatively.<br>-We have added additional language to contextualize the doserate. Thank you for pointing this out!
9	217-218	I have to admit that this level of exposure doesn't seem as though it's anything to be worried about. I know…ALARA…but it's also less than I recorded during a flight from NYC to LAX or from Detroit to Amsterdam.<br>-That is correct that it's low, but it's above standards for exposure to the public, hence our concern. We have modified our language a bit to provide additional context.
9	237	I'm not sure that I agree with the word "hazard" given the low doss reported in this paper. ICRP notes a dose of 2-3 mrem as being a "trivial" dose of radiation and most of the doses reported here are in that range. But even a dose of, say, 1 rem gives only 0.05% risk of developing a fatal cancer – compared to the risk from driving or the background cancer mortality rate, even 1 rem does not seem like much of a "hazard." Is there another word that can be used to indicate that there might be a risk – but that it's very, very low?<br>-We have amended this to state that the risk is low, but in excess of regulatory standards. This better reflects the nature of the risk, as you are correct in that "hazard" conveys too strong of a risk.
		Can the authors include a photograph (or at least a detailed drawing) of their canine whole-body counter (preferably with a canine being counted)?<br>-Due to a camera containing most photos being stolen, we have a fairly limited array of photos showing the system. We have added a figure that shows the device in action, along with a head-on photo of the device. However, the head-on photo of the system unfortunately was taken when one researcher was measuring his own Cs body burden in the system. His backside has therefore been edited out of the photo, which will be mentioned in the caption.

- During neutering surgery, were gross tumor-like growths observed in any part of the body? Did some of the dogs look ''sick''? My question relates to possible radiation-induced soft tissues cancers and/or symptomatic manifestations of osteosarcomas due to internal contamination.

-The animals did not appear “sick” in the overwhelming majority of cases, though the average age of the animals was noted to be rather low. To quote Dr. Betz: “Nothing is noted. However, must take into consideration that it is a very small incision and we reach in with a hook and pull the uterus out so we are not physically exploring the abdomen. There is a possibility of tumors in there, however, highly unlikely at that age.”

- It is stated that ‘’Several animals initially believed to be externally contaminated which did not respond to decontamination efforts were hypothesized to have an internal β-emitter body-burden on bone surfaces which was externally detectable. This was likely due to 90Sr deposited on bone surfaces…’’. However, in one of the boxes of Fig 3a it is reported that 1/288 dogs had contamination that could not be washed off. What is the real number?

Thank you for pointing this out, the number has been revised. Two animals (dogs O007 and O068) were contaminated. Figure 3 has been updated in the manuscript.

- It is not clear the information Fig 2b conveys. Please clarify or simply remove it.

Thank you for pointing this out. We have updated Figure 2 and removed the original Figure 2b.

- Fig 2c: Was the ear tag dosimeter worn only during the TNR procedure? If not, I wonder if the dogs wouldn’t scratch their ears, and some would do so with their contaminated paws and maybe affect the dosimeter reading.

The details about the ear tag study have been removed from the discussion. You are correct that this is a potential confounding factor, but this study is outside the scope of this paper.

- Paraphrasing from the discussion, internal contamination would not pose a health risk to ‘’passersby’’ who would not spend hours interacting with contaminated animals (and therefore exceed the limit of 20 uSv/h). However, what about veterinarians and staff of the TNR program? How long does any procedure take and how many procedures they handle a day?

-Procedures took between 5 and 20min. Depending on dog capture rate and clinic operations, each vet would perform between 10 and 40 procedures per day. Personal dosimeters (RADEX One) were worn by all clinic staff, and total doses did not not exceed 10uSv for any individual for any day. The highest accumulated doses were encountered by staff working in the field, rather than those in the clinic.

No animals surveyed had an external doserate on contact above 1uSv/hr. As a result, the doserates at 30cm are significantly lower than that. I cannot provide doserates at 30cm and 1m without resorting to detailed modeling, as the emitter dimension is of a similar scale to the standoff distance.

-Basic statistical analysis should be provided, as for example to support the statement that internal contamination level correlated with capture location.

Thank you for pointing this out. We have updated the manuscript and removed the problematic statement.

Reviewer #2: Disaster area affected by the nuclear disaster, TNR activities are important due to the overgrowth of animal population and ethical considerations. The authors analyzed internal and surface contamination of dogs protected by TNR and evaluated exposure levels of radioactivity. There are few reports of these activities internationally, and it is useful to disseminate information analyzed by academic organizations internationally. It is worth noting that this study evaluated the risk of contamination by towels and the like contaminated during dog care and the risk to workers, tourists, and other people interacting with the dog. However, unfortunately, detailed data was not shown in the paper, and the details of the data could not be confirmed at the database address shown. In order for this study to provide scientific information to interested readers, it is recommended that the paper provide details of contamination level distribution in the dog populations. Furthermore, from the viewpoint of animal ethics, it is desirable to comprehensively provide information on the health status and infectious diseases of protected dogs. I recommend that author's activities be shared with readers, along with detailed data, statistically analyzed results, and individual health information.

We thank the reviewer for this comment. We apologize for not uploading our materials to the referenced github sooner. All materials have been uploaded to the github including

- A compiled dataset ("data1.xlsx")
- Jupyter notebook for plotting
- Figure components and construction files
- Photos

Reviewer #3: Overall an interesting paper with information that has not previously been reported. However, the paper would benefit from calculations of potential dose to persons handling the dogs - as presented here, it is difficult to see that this would pose an unacceptably high risk. In addition, a photo of the dog-counting setup would be helpful.

We thank the reviewer for this comment. We have updated the manuscript where possible and added more photos in Figure 2 of the dog-counting setup.
