# Supplementary material for: Radioactive contamination in feral dogs in the Chernobyl exclusion zone: Population body-burden survey and implications for human radiation exposure
Source: PLoS One. 2023 Jul 20;18(7):e0283206. doi: 10.1371/journal.pone.0283206 (PMC10358910; doi:10.1371/journal.pone.0283206)
Supplement: Attachment — Submitted filename: Review of Hecla et al.docx [file pone.0283206.s001.docx]

Review of Hecla et al. (Radioactive Contamination in Feral Dogs in the Chernobyl Exclusion Zone: Population Body-Burden Survey and Implications for Human Radiation Exposure)

| **Page** | **line** | **Comment** |
| --- | --- | --- |
|  | Abstract | Even high levels of contamination rarely pose a health risk, even to those who are contaminated. Did the authors quantify the amount of contamination present on any of the dogs, calculate the amount that could reasonably transfer to a person, and determine the potential health effects of this transfer? Barring some sort of calculation to this effect, I have to confess to being somewhat dubious about the potential for external contamination on a dog to transfer to a person in sufficient quantity to pose a health risk. |
| 3 | 30 | Again, I am somewhat dubious about contamination posing a health risk, even if transferred to skin. Can the authors provide some calculations based on average and maximum removeable contamination levels they found, indicating a plausible dose to the skin from incidental contact with contaminated animals, and to the whole body in the event that some of this contamination were to transfer to food and be ingested? I understand that, according to ALARA, any exposure is to be avoided if possible – but I’m also guessing that tourists and animals are likely to be unfamiliar with the ALARA principle , so such an assessment seems reasonable. |
| 4 | 34 | Similarly, can the authors calculate exposure to people at a distance of, say, 30 cm and 1 m from the contaminated animals they measured? How does the calculated dose rate compare to the actual dose rates measured? |
| 4 | 75 | How does the background noted here compare to unshielded background count rate outside at this location? |
| 5 | 96 | Do the authors have a citation for the high counting efficiency noted here? It’s not that I doubt them – just that Knoll notes an intrinsic counting efficiency of a little less than 90% for a 4” NaI detector and, when we add to that a geometric counting efficiency of 50% or less, the absolute counting efficiency is more likely around 40-45% for Cs-137. |
| 5 | 100 | Good call on not using K-40! The amount in concrete (and brick, for that matter) depends strongly on the clay mineralogy of these materials |
| 6 | 131 | Can the authors provide a reference (or calculation) for 22% counting efficiency for Sr-90? Also, is this for Sr-90 alone, or are you assuming that Y-90 is in equilibrium? If Y-90 is present, the actual counting efficiency might be much higher due to the presence of the second beta and its much higher energy. |
| 7 | 160 | How did the authors differentiate between the beta and gamma radiation when determining the counting efficiency? Or is the gamma counting efficiency simply too low to be a factor (in which case, perhaps this can be noted)? |
| 7 | 168 | Here, too, calculated exposure rates would be helpful. |
| 8 | 190 | Do the authors recommend that anyone coming into physical contact with animals wear gloves, wash their hands, shower, change clothing, etc afterwards to reduce their exposure? |
|  |  | Can the authors suggest mechanisms by which touching a contaminated animal can result in significant internal contamination and likely exposures? I can think of a few (e.g. eating a sandwich before washing one’s hands) – but this seems unlikely to transfer sufficient contamination to produce a high internal exposure. |
| 8 | 200 | The paper mentions using an “energy-compensated NaI(Tl) detector. I am familiar with using energy-compensated GM detectors and have used many of them over the years. But I have never heard the term applied to sodium iodide. Can the authors indicate which of the NaI(Tl) detectors mentioned was energy-compensated? |
| 8 | 201 | Thanks for noting that 1 µSv/hr is not a very high dose rate – this might be worth mentioning in the abstract as well, even if only qualitatively. |
| 9 | 217-218 | I have to admit that this level of exposure doesn’t seem as though it’s anything to be worried about. I know…ALARA…but it’s also less than I recorded during a flight from NYC to LAX or from Detroit to Amsterdam. |
| 9 | 237 | I’m not sure that I agree with the word “hazard” given the low doss reported in this paper. ICRP notes a dose of 2-3 mrem as being a “trivial” dose of radiation and most of the doses reported here are in that range. But even a dose of, say, 1 rem gives only 0.05% risk of developing a fatal cancer – compared to the risk from driving or the background cancer mortality rate, even 1 rem does not seem like much of a “hazard.” Is there another word that can be used to indicate that there might be a risk – but that it’s very, very low? |
|  |  | Can the authors include a photograph (or at least a detailed drawing) of their canine whole-body counter (preferably with a canine being counted)? |
